# Colic anastomotic leakage risk factors

**Published:** 2013-12-25

**Authors:** MD Calin, C Bălălău, F Popa, S Voiculescu, RV Scăunașu

**Affiliations:** * Department of General Surgery, "Carol Davila" University of Medicine and Pharmacy,„Sf. Pantelimon” Emergency University Hospital, Bucharest; ** Department of General Surgery, "Carol Davila" University of Medicine and Pharmacy, Coltea University Hospital, Bucharest

## Abstract

Abstract

Background: Anastomotic leakage is a dreaded complication of colorectal surgery, as it greatly increases the morbidity, mortality and has been associated with augmented local recurrence and diminished survival.

The frequency of this complication is high in emergency colorectal surgery, especially for bowel occlusion, (13% for emergency vs. 4% in elective), due to visceral distension and, therefore, an incongruence in the size of each of the stumps, combined with the lack of mechanical preparation and risk of fecal contamination during operation.

Methods: We studied the incidence of anastomotic fistula in the surgery clinic of the “Sf. Pantelimon” Emergency Hospital, between 2006 and 2010, on a lot of 251 patients who underwent different types of colic resection.

Apart from the anatomic location of the disease, and the level of anastomosis, we included in our database the following criteria: the patient’s age and gender, type of colic pathology, surgical technique, emergency or elective surgery, comorbidities.

Results: An ileocolic anastomosis was performed for 84 patients (33,46 %), for 114 patients (45,41%) a colo-colic anastomosis was carried out, 2 patients (0,79%) had ileorectal anastomosis and 51 patients (20,31%) underwent a colorectal anastomosis.

From the comparative analysis of risk factors (the emergency interventions, the anastomosis location, the age and gender of the patient), a significantly increased value of the relative risk of anastomotic fistula was registered for the cases with emergency intervention (x 6,61) and for the colorectal anastomosis following the left hemi colectomies (x 2,23).

Discussions: In our study, among the clinical and biological factors analyzed, emergency intervention was the most signiﬁcant factor associated with anastomotic leakage. Surgery performed in emergency settings, on debilitated patients without adequate preoperative preparation, has an increased risk for anastomotic dehiscence.

## Introduction

Anastomotic leakage is a dreaded complication of colorectal surgery after colorectal surgery as it greatly increases the morbidity, mortality and has been associated with high local recurrence and diminished survival after colorectal cancer surgery [**1**].

The leak incidence varies from 3.4% to 40% (the larger figures include subclinical radiological diagnoses) [**1**]. The morbidity and mortality related to anastomotic breakdown is considerable. Fielding in his study showed that among 1466 patients who underwent large bowel anastomosis, the mortality for the group with an anastomotic leak was of 22% compared to 7.1% for the patients without a leak [**2**].

The frequency of this complication is high in emergency colorectal surgery, especially for bowel occlusion, (13% for emergency vs. 4% in elective), due to visceral distension and, therefore, an incongruence in the size of each of the stumps, combined with the lack of mechanical preparation and risk of fecal contamination during operation [**3**].

Many factors may play a pathological causative role, including: contribution from faulty technique, ischemia of the intestine at the suture line, excessive tension across anastomosis or mesentery, obstruction distal to the anastomosis or the presence of local sepsis.

**Table 1:** The safety of the planned anastomosis can be questioned in the presence of any of these risk factors [**4**, **5**]

Anastomotic leak risk factors
Surgeon-Related Factors (Wrong Intraoperative Judgment, Poor Surgical Technique)
Local Complications (Sepsis, Bowel Preparation, Drains, Role Of Omentum And Peritoneum, Anesthetic Drugs, Protective Stoma)
Systemic Complications (Nutritional Status, Blood Loss)

The principles of the good and reliable colorectal anastomosis are the following: 1. good exposure and access to large bowel (long enough incision) 2. adequate blood supply of anastomosed stumps, 3. prevent sepsis or gross fecal contamination 4. sutures or staplers should be properly placed assuring good approximation of all layers of bowel wall (most important is sub mucosa) 5. no tension of the anastomosis (always release the splenic flexure in left colorectal surgery) 6. check for distal obstruction 7. The patient should be well nourished and the large bowel should be mechanically well prepared (no fecal contamination) [**6**, **7**].

Blood supply is essential for the proper healing of anastomosis. The cut ends of bowel should bleed. To assess the adequate blood supply of the bowel stump the routine measurement of tissue oxygen and laser Doppler flowmetry are currently being evaluated in many centers. Micro vascular disease, as seen along with hypertension and smoking, was reported to predispose patients to anastomotic leakage [**8**].

**Systemic factors**

The role of systemic factors in the etiology of anastomotic leak is not yet completely defined. Among systemic factors, at least three of them do seem to play a significant role and they are the following: malnutrition (serum albumin level below 3.0 g/dl), anemia and excessive blood loss, and advanced malignancy.

Excessive blood loss results in the reduction of colonic blood flow with subsequent tissue ischemia. Bloodloss inevitably leads to the need of transfusion, which in turn has been shown to decrease the patients’ immunocompetence.

**Nutrition**

Preoperative albumin is a known prognostic indicator of postoperative complication. Many studies have indicated an important association between low serum albumin and excess mortality in healthy populations of both western and eastern countries [**9**, **10**]. In a multivariate analysis, low serum albumin level (3.5 g/dL) was significantly associated with anastomotic leaks. Whether it is hypoalbuminemia or hypoproteinemia, evidence of protein deficiency places a patient at a higher risk for anastomotic leaks. Preoperative maximization of nutrition is an important risk-reduction measure.

**Surgical technique**

When it comes to bowel anastomosis, the need for good edge-to-edge apposition and adequate luminal patency is self-evident.

In relation to efficacy, applicability, and safety, it has been demonstrated that the use of surgical stapling instruments is comparable to that of conventional suturing methods. In certain situations, staplers offer the facility to achieve reconstructions that would be difficult to be accomplished manually, and their popularity in that setting seems justifiable.

**Table 2:** Comparison of stapled versus sutured colorectal

Investigators	Number of patients	Staple technique leak rate (%)	Suture technique leak rate (%)	p
Docherty et al[**5**]	732	4.7	4.3	0.93
Fingerhut et al[**9**]	113	13	18.7	0.05
Everett et al[**13**]	100	0	2	NS
Demetriades et al[**14**]	207	6.3	7.8	0.69

A meta-analysis in 1998, concluded that there is no difference between hand-sewn and stapled anastomosis for the majority of outcome measures including mortality, leak rates, local cancer recurrences, and wound infections.

**Table 3:** Risk factors – anastomotic leak

Risk factors for development of an anastomotic leak (1726 patients)
Pre-existing sepsis vs. no-sepsis (5.0% vs. 1.5%,p<0.005)
Heavy intraoperative fecal contamination vs. minimal contamination (11.3% vs. 23%,p<0.001)
Per anastomotic drains vs. no drain (6.3% vs. 1.9%, p<0.001)
Proximal diverting stoma vs. no stoma (9.8% vs. 1.9%,p<0.001)
Emergency vs. elective surgery (4.1% vs. 1.9%, p - ns)

**Defunctioning ileostomy**

A prospective study in 2005 concluded that a defunctioning ileostomy has no influence over whether a low rectal anastomosis will leak after surgery. Rather, it minimizes the sequel of a leak in high-risk patients. It has significant morbidities and should therefore be used judiciously [**11**].

## Methods

Due to their interdependency, no consensus regarding the role that each factor plays in the anastomosis healing process has been accepted. Therefore, a better knowledge of risk factors is of a decisive importance, especially with regard to the consequences for perioperative management or tactical considerations of surgical procedures.

 The aim of this study was to evaluate the possible risk factors for anastomotic leak development after colorectal cancer excision, and also to determine the predictive value of each independent risk factor identiﬁed.

We studied the incidence of anastomotic fistula in the surgery clinic of the Saint Pantelimon Emergency Hospital Bucharest, between 2006 and 2010, on a lot of 251 patients who underwent different types of colic resection.

Apart from the anatomic location of the disease, and the level of the anastomosis, we included in the database the patient’s age and gender, the type of colic pathology, the surgical technique, emergency or elective surgery, comorbid conditions, and the anesthetic and surgical risks.

An ileocolic anastomosis was performed for 84 patients (33,46%), for 114 patients (45,41%) a colocolic anastomosis was carried out, 2 patients (0,79%) had ileorectal anastomosis and 51 patients (20,31%) underwent a colorectal anastomosis.

**Fig. 1:**
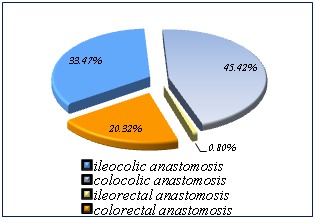
Ileocolic anastomosis, colocolic anastomosis, ileorectal anastomosis, colorectal anastomosis

The assessment of the different risk factors involvement on the occurrence of anastomotic fistula was performed through the calculation of the relative specific risk (Table 1 and the displayed formula), on the two groups: the one of the patients with a simple postoperative evolution, without anastomotic fistula – 230 cases (the control group) and the group of patients who developed a postoperative anastomotic fistula – 21 cases (the test group).

## Results

1.The relative risk of anastomotic fistula in the cases where there is an emergency surgical intervention was 6,61 times higher than in the cases where the intervention was scheduled.

2.The analysis of the relative risk of fistula in relation to the level of anastomosis revealed a value of only 1.16 times higher for ileocolic anastomosis in relation to the colocolic one, and 2,23 times higher of fistula formation for the colorectal one in relation to the colocolic anastomosis. With regard to the relative risk of ileorectal anastomosis of fistula generating, the small number of cases cannot induce a statistically significant result.

3.The relative risk of fistula calculated for the older patients (over 65 years old) compared to the one below 65 years old is 1,31 times higher.

**Fig. 2:**
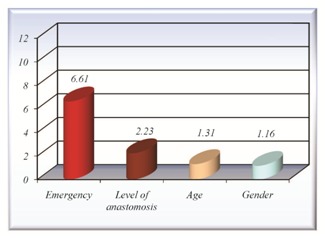
The relative risk of fistula

## Discussions

Suture dehiscence has been associated with one ﬁfth to one third of all postoperative deaths in patients who underwent an intestinal anastomosis [**12**].

Several studies regarding factors that contribute to failure of large bowel anastomosis have been published. The majority of these studies had focused on the anastomosis leakage after cancer resections. Furthermore, there are no reports examining the predictive value of each independent factor for anastomotic leak development.

Premorbid medical conditions reﬂect the patient’s general status and add to the surgical risk, affecting anastomotic healing [**12**]. Only smoking, cardiovascular disease, lung disease, diabetes, preoperative anemia (serum hemoglobin level ≤11 g/dl) and preoperative hypoproteinemia (total serum protein level ≤6 g/dl) were signiﬁcantly associated with an anastomotic leak occurrence. Multivariate analysis showed that serum hemoglobin level less than 11 g/dl and serum protein level less than 6 g/dl were independent risk factors for anastomotic leakage [**13**,**14**].

Another multivariate analysis showed that the American Society of Anesthesiologists Grade III to V (P = 0.04; odds ratio, 5.6; 95 percent confidence interval, 1.6-15.3) and emergency operation (P = 0.03; odds ratio, 4.6; 95 percent confidence interval, 1.9-9.8) were independent factors associated with anastomotic leakage.

The risk of anastomotic leakage was of 8.1% (odds ratio, 10.5; 95% confidence interval, 2.7-26.8) if both factors were present [**15**,**16**].

In our study, among the clinical and biological factors analyzed, the emergency intervention was the most signiﬁcant factor associated with anastomotic leakage. Surgery performed in an emergency setting, on debilitated patients without adequate preoperative preparation, has an increased risk for anastomotic dehiscence.
